# Movement Disorders Related to Gluten Sensitivity: A Systematic Review

**DOI:** 10.3390/nu10081034

**Published:** 2018-08-08

**Authors:** Ana Vinagre-Aragón, Panagiotis Zis, Richard Adam Grunewald, Marios Hadjivassiliou

**Affiliations:** Academic Department of Neurosciences, Sheffield Teaching Hospitals NHS Foundation Trust, Sheffield S10 2JF, South Yorkshire, UK; takiszis@gmail.com (P.Z.); Richard.Grunewald@sth.nhs.uk (R.A.G); m.hadjivassiliou@sth.nhs.uk (M.H.)

**Keywords:** movement disorders, coeliac disease, gluten, gluten free diet

## Abstract

Gluten related disorders (GRD) represent a wide spectrum of clinical manifestations that are triggered by the ingestion of gluten. Coeliac disease (CD) or gluten sensitive enteropathy is the most widely recognised, but extra-intestinal manifestations have also been increasingly identified and reported. Such manifestations may exist in the absence of enteropathy. Gluten sensitivity (GS) is another term that has been used to include all GRD, including those where there is serological positivity for GS related antibodies in the absence of an enteropathy. Gluten ataxia (GA) is the commonest extraintestinal neurological manifestation and it has been the subject of many publications. Other movement disorders (MDs) have also been reported in the context of GS. The aim of this review was to assess the current available medical literature concerning MDs and GS with and without enteropathy. A systematic search was performed while using PubMed database. A total of 48 articles met the inclusion criteria and were included in the present review. This review highlights that the phenomenology of gluten related MDs is broader than GA and demonstrates that gluten-free diet (GFD) is beneficial in a great percentage of such cases.

## 1. Introduction

The term Gluten related disorders (GRD) covers a broad spectrum of immune-mediated clinical manifestations that are triggered by the same environmental insult; dietary gluten. Coeliac disease (CD) or gluten-sensitive enteropathy is the best-characterized disease within this wide clinical spectrum. Moreover, CD is the most common immune-mediated gastrointestinal (GI) disorder diagnosed both in childhood and adulthood, with an increasing global prevalence [1].

The classical presentation of CD includes GI symptoms (i.e. diarrhoea, abdominal pain, abdominal bloating), malnutrition, anaemia, and weight loss. The treatment is strict adherence to a gluten-free diet (GFD). Rarely patients with CD may no longer respond to GFD and are diagnosed with refractory CD a condition that may require immunosuppressive treatment [2].

Often, patients with gluten sensitivity (GS) can present with subtle or even no GI symptoms and a wide range of extra-intestinal manifestations affecting different organs. Gluten ataxia (GA), defined as sporadic cerebellar ataxia in the presence of circulating antigliadin antibodies and no alternative etiology for the ataxia, is by far the commonest neurological presentation. Nonetheless, several other neurological manifestations have been reported, such as epilepsy [3,4], gluten encephalopathy [5,6], myopathy [7], peripheral neuropathy [8,9,10,11], and other movement disorders (MDs). On some occasions, MDs have been recognized as a complication of or co-existing with systemic autoimmune diseases [12,13].

In this paper, we performed a systematically review of the current medical literature on MDs in CD and GS. We have excluded GA as this has been extensively studied and reviewed previously, and as such, it is a well-recognised entity [14,15].

## 2. Materials and Methods

### 2.1. Literature and Search Strategy

A systematic computer-based literature search on the topic was conducted on February 12th, 2018 using the Pubmed database. For the search we used two Medical Subject Headings (MeSH) terms in all fields. Term A was “celiac” or “coeliac” or “gluten” and term B was “chorea” or “choreiform” or “choreic” or “choreoathetosis” or “athetosis” or “tremor” or “dystonia” or “hemidystonia” or “torticollis” or “antecollis” or “anterocollis” or “retrocollis” or “laterocollis” or “blepharospasm” or “ballism” or “hemiballism” or “ballismus” or “hemiballismus” or “stiff” or “Parkinson” or “Parkinson’s” or “parkinsonism” or “myoclonus” or “myoclonic” or “tic” or “myokymia” or “myorhythmia” or “Huntington” or “Huntington’s” or “dyskinesia”, and “RLS”. Limitations included English language, human species, and full text available. We also perused the reference lists of the papers since the drafting of this paper in order to identify papers not identified through the search strategy.

### 2.2. Inclusion and Exclusion Criteria

To be included in this review, the articles had to meet the following inclusion criteria:

1. To be original clinical papers.
2. To study human subjects.
3. To involve single cases, case series, or retrospective observational studies with the combination of CD or GS and MDs.

Exclusion criteria included:

1. Reviews, book chapters, letters to editors, and editorials that are not providing new data.
2. Papers referring only to GA.

## 3. Results

### 3.1. Search Results

**Figure 1:**
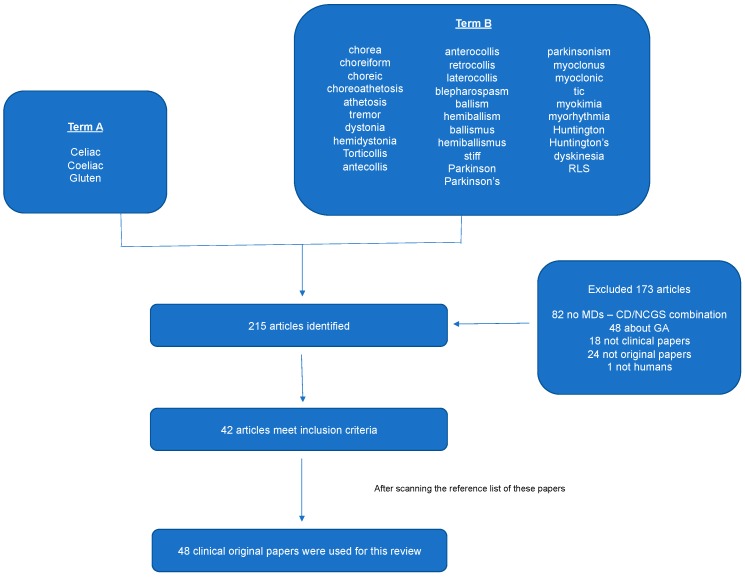
PRISMA chart. To be included in this review, the articles had to meet the following inclusion criteria: (1) To be original clinical papers, (2) to study human subjects, (3) to involve single cases, case series or retrospective observational studies with the combination of Coeliac disease (CD) or gluten sensitivity (GS) and movement disorders (MDs).

The search strategy that is described above resulted in the identification of 215 articles. After the eligibility assessment, 173 articles were further excluded, as they did not meet our inclusion criteria. Scanning the reference list, six more papers were identified. In total, 48 papers were used for this review. Table 1 summarizes the characteristics of these papers and Figure 1 illustrates the study selection process. Table 2 summarizes the characteristics of the patients and the response to GFD in each movement disorder type.

### 3.2. Chorea

Chorea is defined as irregular, brief, purposeless movements that flit from one body part to another, and it can be inherited or acquired [16,17]. Vascular, drug-induced, AIDS-related, and metabolic were the most common causes of acquired chorea in the case series published by Piccolo et al. [18] Investigation of chorea should be directed to the most likely causes [19].

The first report suggesting the link between CD and chorea was by Willis and colleagues, who conducted a retrospective observational study to investigate patients with dermatitis herpetiformis (DH), which is another extraintestinal manifestation of CD affecting the skin, for evidence of neurological manifestations [20]. One out of 35 patients with DH suffered chorea. However, of note is that this patient had been on phenytoin over the last 14 years after a single seizure that may have played a role in the development of chorea [21,22]. However, subsequently, case reports and small cases series of patients with chorea and CD or GS, and no other risk factors for developing chorea, have been published [23,24,25,26].

Demographic data of patients with gluten related chorea were available in seven of the cases. The choreiform movements were described as generalized affecting predominantly the upper limbs. The majority was female (86%) and their mean age of onset was 57.4 ± 12.9 years. Five patients had biopsy proven CD, whereas two patients had only serological evidence of GS. HLA DQ2 was tested in four of the patients, and positive just in two of the cases tested. There was a significant improvement in the choreiform movements after embarking on a GFD in five of the patients and no response in two.

### 3.3. Restless Legs Syndrome

Restless leg syndrome (RLS) is a circadian disorder appearing typically at the end of the day, being characterised by an intense and irresistible urge to move the lower extremities, either by itself or in response to unpleasant leg sensations. Symptoms typically improve while walking, stretching, or moving the lower limbs [27]. There are five essential diagnostic criteria and all must be met: (1) An urge to move the legs usually but not always accompanied by or felt to be caused by uncomfortable and unpleasant sensations in the legs, (2) Symptoms begin or worsen during periods of rest or inactivity such as lying down or sitting, (3) Symptoms are partially or totally relieved by movement, such as walking or stretching, at least as long as the activity continues, (4) Symptoms only occur or are worse in the evening or night than during the night, and (5) The occurrences of the above features are not solely accounted for as symptoms primary to another medical or behavioral condition [28]. The prevalence of RLS varies among different population surveyed. The data from REST (RLS Epidemiology, Symptoms, and Treatment), which is the largest trial till date with 23.052 patients, revealed that any degree of RLS symptoms was present in 11.9% [29]. The pathogenesis of RLS continues to be only partially understood, but there is substantial evidence for abnormalities in brain iron metabolism and dopaminergic dysfunction probably plays a key role [30]. RLS severity increases with decreased peripheral iron [31]. In fact, its prevalence is significantly greater in individuals with iron-deficiency anaemia [32].

Whether there is a link between RLS and CD or GS remains controversial. The first report suggesting an association between CD and RLS was by Manchanda et al. who presented a consecutive case series of four patients with RLS, low serum ferritin, and biopsy proven CD [33], which was considered to be the underlying cause for low serum ferritin. Subsequently, in two studies RLS was found to be more frequent in patients with CD than in controls [34,35]. On the other hand, however, Cikrikcioglu et al. studied the presence of antibodies relating to GS (tissue transglutaminase antibody IgA and IgG, antiendomyisium antibody IgA and IgG, and/or antigliadin antibody IgA and IgG) in 96 patients with RLS and age, sex, and BMI matching 97 subjects without RLS and could not demonstrate a significant difference between the two groups [36]. Furthermore, contradictory data have hitherto been published related to iron metabolism parameters in coeliac patients with active RLS and coeliac patients without RLS. Weinstock et al., found that concomitant iron deficiency was significantly more common in coeliac patients with RLS than in coeliac patients without RLS, but there were no statistically significant differences in haemoglobin levels between both groups [34]. In contrast, Moccia et al. could not find statistically significant differences in blood levels of iron, ferritin, and MCV between coeliac patients with RLS and coeliac patients without RLS in their study. However, haemoglobin levels were significantly lower in coeliac patients with RLS than in coeliac patients without RLS [35].

There are no data available regarding the age of onset of RLS in patients with CD or GS. The majority of described patients are female (91%). All of the patients had biopsy proven CD. The information about response to GFS is limited; three out of four CD patients with RLS improved on GFD and iron supplementation, whereas one patient improved after being on a GFD without receiving iron supplementation and still having low ferritin levels [33]. Weinstock and colleagues reported that 50% of the CD patients found relief in their RLS symptoms being on GFD, and similarly not all were receiving iron supplementation [34], suggesting that GFD can independently improve the RLS symptoms in people with RLS and CD or GS.

### 3.4. Myoclonus

Myoclonus is defined as a sudden, brief, shock like involuntary movement caused by active muscle contraction (positive myoclonus), or inhibition of on-going muscle activity (negative myoclonus) [37]. All of the studies that attempt to evaluate the general occurrence of myoclonus have various biases. There is an epidemiological study on myoclonus due to any cause in a defined population where the average annual incidence of pathological and persistent myoclonus for 1976 to 1990 was 13 cases per 100,000 person-years [38]. Progressive myoclonic ataxia (PMA) is a rare syndrome where progressive myoclonus and cerebellar ataxia coexist [39].

The first report suggesting the comorbidity of CD and PMA was by Cook and colleagues in 1966 [40]. Subsequently, several case reports were published [41,42,43,44,45,46,47]. Lu and colleagues published a case series of patients with ataxia and myoclonus providing for the first time electrophysiological evidence for the cortical origin of the myoclonus [48]. These findings were further confirmed later in many cases and case series [49,50,51,52,53]. The largest case series was published by Sarrigiannis et al., and included nine patients with CD, myoclonus of cortical origin and ataxia. All of the patients were compliant with a strict GFD, as evident by the elimination of gluten-related antibodies. Nonetheless, there was still evidence of enteropathy in all, and in some it was suggestive of refractory CD type 2. Aggressive immunosuppression improved ataxia and enteropathy in contrast to myoclonus that remained unchanged [54].

The mean age of onset of gluten related PMA is 47.7 ± 17.3 years. The majority of patients are males (55%). All of the patients reported to date had biopsy proven CD. Myoclonus phenomenology was described as often stimulus sensitive, asymmetrical, and irregular, generally focal at onset involving one or more limbs and sometimes the face, with a tendency to become gradually more generalized. However, it tends to still remain asymmetrical. In general, GFD, even in combination with aggressive immunosuppression, shows minimal effect on the myoclonus, but it may improve the enteropathy and the ataxia.

### 3.5. Palatal Tremor

Palatal tremor is defined as brief, rhythmic involuntary movements of the soft palate. It can be divided into symptomatic palatal tremor (SPT) and essential palatal tremor (EPT). SPT results from an insult in the Mollaret triangle being composed of the inferior olive, red nucleus, and contralateral dentate nucleus. In contrast, in EPT, no lesion is demonstrable. Data regarding the prevalence of SPT or EPT are scarce. SPT rarely can be associated with ataxia and is referred as progressive ataxia palatal tremor syndrome (PAPT) [55].

To date, three case reports of PAPT (one male and two females) in the context of CD have been reported [41,50,56]. The mean age of onset was 51.3 ± 8.1 years. HLA DQ2 was tested just in one of the cases and was positive. Two of the patients had biopsy proven CD, whereas another refused biopsy but it was diagnosed with GS based on the high titer of antigliadin antibodies. In the latter, palatal tremor improved after GFD, whereas no response to GFD was evident on the other two cases.

### 3.6. Dystonia

Dystonia is defined as a hyperkinetic movement disorder characterized by sustained or intermittent muscle contractions that cause abnormal involuntary repetitive movements, postures, or both [57]. There is significant variability in the reported prevalence of dystonia because to date the epidemiological studies published have adopted different methodologies for case ascertainment. A systematic review and meta-analysis that was published in 2012 reported a prevalence of 16.43 per 100,000, but it is likely to be underestimated, with many cases remaining undiagnosed [58]. The pathophysiology of dystonia is still poorly understood [59].

Two isolated cases of patients (one male and one female) with previous biopsy proven CD diagnosis that presented with dystonia have been reported to date [51,60]. The mean age of onset was 49.5 ± 2.1 years. In both cases, dystonia was focal affecting one limb. There was no response to GFD. In a large study where Bürk et al. screened patients with biopsy proven CD for neurological symptoms or signs, 3 out of 72 patients presented with dystonia [61]. Wittstock and colleagues reported a case of secondary dystonia due to cerebral vasculitis in a patient with biopsy proven CD [62]. This case may only illustrate coincidence of isolated vasculitis and CD. However, dystonia due to vascular lesion in the context of vasculitis and CD were diagnosed simultaneously and the dystonic symptoms improved after being combined with GFD and immunosuppressive therapy. This led the authors to postulate a causative relationship between the dystonia and CD.

### 3.7. Postural Tremor

Tremor is a rhythmic oscillation of a body part, which is produced by either alternating or synchronous contractions of reciprocally innervated antagonist muscles [63]. Several cases of patients with CD presenting with tremor often in association with or without later development of ataxia have been reported [64,65,66,67]. Tremor is focal and generally postural, affecting mainly the limbs, but also head, jaw, and tongue. 

The mean age of onset of tremor is 54.6 ± 14.9 years and the male:female ratio was 1:2. All of the patients had biopsy proven CD and 67% had HLA DQ2 positive. There was a significant response to GFD in two-thirds of patients. Of interest is that postural tremor of abrupt onset has also been reported even in childhood in a case of a four year old boy with CD who suffered central pontine myelinolysis without electrolyte abnormalities [68]. The lack of neurophysiological characterisation of the tremor in such reports means that it is not possible to distinguish from myoclonus.

### 3.8. Stiff-Person Syndrome

Stiff person syndrome (SPS) is characterised by the increased tone of axial and limb muscles, with superimposed muscle spasms leading to lumbar hyperlordosis, impaired gait, falls, and autonomic dysfunction associated with anti-GAD and/or other autoantibodies [69]. This syndrome has a strong concurrence with other autoimmune entities [70,71,72]. SPS has an estimated prevalence of 1–2 cases per million with an incidence of one case per million per year [73]. In their study, Hadjivassiliou et al. screened patients with neurological disorders of unknown aetiology for GS and showed that such patients had a higher prevalence of circulating antigliadin antibodies [74]. In particular, out of 131 patients with GS and neurological disorders of unknown aetiology, four had the diagnosis of SPS [75]. A higher prevalence of GS in patients with SPS was found than what would be expected in the context of coexistence of two autoimmune diseases [76]. As SPS symptoms follow a relapsing-remitting pattern, the assessment of responsiveness to GFD is challenging. Nevertheless, there is evidence of reduction of the anti-GAD antibody titer after the implementation of GFD suggesting that GFD may be beneficial in treating the condition [77].

### 3.9. Parkinsonism

Parkinsonism is defined as a hypokinetic syndrome and it is characterised by the presence of resting tremor, rigidity, bradykinesia, and postural instability. The most common primary cause of parkinsonism is idiopathic Parkinson’s disease (IPD), with a prevalence of 130 per 100,000 [78], but many secondary or acquired causes of parkinsonism exist [79]. Recently, Di Lazzaro and colleagues reported a case of improvement of parkinsonian symptoms after GFD implementation in a patient with biopsy proven CD [80]. Gonzalez Aleman and colleagues presented the case of a patient with parkinsonism and increased echogenicity in substantia nigra that is associated with biopsy proven CD and clozapine treatment. They postulated that the patient may have had subclinical IPD, which was unveiled after clozapine exposure or that she had a neuroleptic-induced akinetic rigid syndrome. However, they also speculated that CD might have played a role in the pathogenesis, taking into account the young age of the patient [81].

In the study of neurological disorders in a group of unselected patients with biopsy-proven CD conducted by Bürk and colleagues, 2 out of 72 patients fulfilled the diagnostic criteria for PD [61]. However, as these patients had previously followed a GFD and they were considered to be in remission, this finding may have just merely been coincidental. One out of the 10 CD cases reported by Luostarinen and colleagues, which were initially referred to the neurological department because of neurological symptoms and were finally found to have CD, presented with a four-month history of an asymmetrical left sided parkinsonian syndrome. Four years later diagnosis of CD was established, but the patient was never compliant with GFD [65]. In all cases, parkinsonism was described as affecting one side of the body more than the other. The mean age of onset was 54.0 ± 18.7, all the patients were females and had a biopsy proven CD. Only one patient out of three showed a response to GFD. Given that Parakinsonism is a relatively common neurological condition, the co-occurence of CD and Parkinsonism may well be co-incidental.

### 3.10. Tics

Tics are sudden, rapid, non-rhythmic, intermittent muscle movements (motor tics), or sounds (phonic tics), which can be classified as simple or complex [82,83]. What characterizes tics is an inner urge to make the movement or a local premonitory sensation experienced and temporarily relieved by its performance. Several studies have examined the prevalence of tic disorders. However, wide variation was evident across these studies in terms of specific diagnoses examined and the age of the population under study [84]. Zelnik and colleagues conducted a study to look for a broader spectrum of neurologic disorders in CD. However, an association between CD and Tic disorders was not demonstrated [85]. In the previously mentioned study that was conducted by Bürk and colleagues, two out of 72 suffered with Tics [61].

Gilles de la Tourette syndrome (GTS) is characterised by the presence of multiple motor tics and one vocal or phonic tic persisting for more than a year, from the appearance of the first tic [86]. A case report of a patient with CD, HLA DQ8 positive, and GTS has been reported and it was shown that GFD could be beneficial in managing the tics [87]. Rodrigo et al. carried out a prospective interventional study to analyse and evaluate the efficacy of GFD in a series of childhood and adult patients with GTS. Gluten removal was useful for reducing the intensity and frequency of motor and vocal/phonic tics and OCD symptoms [88].

### 3.11. Other Movement Disorders

#### 3.11.1. Opsoclonus-Myoclonus

Opsoclonus-myoclonus syndrome is characterised by opsoclonus, myoclonus, and ataxia, associated with behavioural changes [89]. Opsoclonus is encompassed in the group of eye movement abnormalities known as saccadic intrusions, defined as involuntary multidirectional saccades that interrupt steady fixation. [90,91]. Deconinck and colleagues reported the case of a two-year-old male with CD, cerebellar ataxia, palpebral flutter, action myoclonus and opsoclonus. Both GI symptoms, as well as the neurological symptoms, improved after GFD implementation and treatment with steroids and immunoglobulins [92].

#### 3.11.2. Propiospinal Myoclonus

Propiospinal myoclonus (PSM) is an uncommon movement disorder involving axial muscles characterized by painless, usually flexor arrhythmic jerks affecting the trunk, hips, and knees. It is often stimulus sensitive and typically worsens while adopting supine position [93]. The etiology of PSM is most commonly idiopathic [94]. Zhang and colleagues reported a case of a 23-year-old lady who developed PSM in the setting of CD [95]. On examination there were continual relatively rhythmic flexor muscle jerks affecting the neck, shoulders, trunk and hips. The jerks were elicited by patellar tendon tap in the supine position but not while sitting. The myoclonus began minutes to hours after gluten intake. There was complete resolution of the symptoms on GFD.

#### 3.11.3. Paroxysmal Dyskinesia

Paroxysmal dyskinesia is defined as a group of episodic abnormal involuntary movements manifested by recurrent attacks of dystonia, chorea, athetosis, or a combination of these disorders [96]. Most cases are familial and usually autosomal dominant, but some are idiopathic [97]. Hall and colleagues reported the case of a female patient with abnormal movements from the age of six months [98]. The episodes were described as twisting of her upper body to one side, with an outstretched arm as well as a flexed position of the left leg lasting from 5 to 30 min and appearing several times in a day. At the age of eight, she presented with GI and after extensive workup, the diagnosis of biopsy proven CD was established. She was commenced on GFD and the symptoms resolved completely after six months.

#### 3.11.4. Myorhythmia

Myorhythmia is characterised by slow rhythmic movements, usually involving the limb or cranial muscles, and it has been linked with a variety of identifiable etiologies [99]. Dimberg and colleagues reported the case of a 68-year-old lady with refractory CD who presented with myorhythmia [100] of the tongue, cheek, and fingers. Movements were described as continuous, synchronous, semirhythmic contractions occurring at rest as well as with movement. Two months after the onset of myorhythmia, the patient developed an encephalopathy that was confirmed by neuroimaging and neuropathology, that appeared to be inflammatory. An infectious aetiology was excluded by CSF analysis. Screening for autoimmune encephalitis and paraneoplastic syndromes was negative. The patient was HLA-DQ2 and -DQ8 positive.

#### 3.11.5. Myokymia

Myokymia is characterised by spontaneous, fine fascicular contractions of muscle that usually can be seen on the skin as vermicular or continuous rippling movements [101]. A case of a 72-year-old lady with biopsy-confirmed CD, who initially presented with progressive generalized myokymia has been reported [102]. On examination, prominent vermicular, undulating slow movements of her orbicularis oris, mentalis, and right intrinsic hand muscle were noticed. EMG revealed myokymic potentials in several muscles. Subsequently, she developed both, action and stimulus-sensitive myoclonus, as well as ataxia. There was histologic improvement on jejunal biopsy on GFD. In contrast, there was no clinical progression of neurologic symptoms.

## 4. Conclusions

This paper aimed to systematically review the current literature regarding MDs in CD and GS. To our knowledge, this is the first review on the topic highlighting that the phenomenology of the gluten related movement disorders is broad and that GFD is apparently beneficial in many cases. Our review also indicates the following key points:

1. GS and CD should be considered in the diagnostic workup of MDs of unknown etiology in patients of all ages and both genders, even in the absence of GI symptoms.
2. Neurologic manifestations, including MDs, may precede the diagnosis of GS and CD.
3. Some of the MDs may improve or resolve after dietary gluten removal, so early diagnosis should rapidly lead to the implementation of GFD.
4. Once GFD is implemented, it should generally continue lifelong like in CD. In fact, in some cases, sporadic accidental gluten ingestion continues to trigger the MD.
5. In contrast, other types of MDs, such as ataxia with myoclonus, appear to be linked to refractory CD and when observed, there is prompt need for repeat biopsy of the small intestine and often aggressive immunosuppression.
6. The fact that the majority of the included papers refer to CD rather than the broader spectrum of GS may mean that the relationship of MDs to GS without enteropathy is under-studied.

## Figures and Tables

**Table 1:** Characteristics of the papers included in the review.

**Number of Papers Related to Each Movement Disorder (%)**	
Chorea	5 (11%)
Restless leg syndrome	4 (8%)
Myoclonus	15 (31%)
Palatal tremor	3 (6%)
Dystonia	3 (6%)
Tremor	5 (11%)
Stiff Person Syndrome	2 (5%)
Parkinsonism	3 (6%)
Tics	3 (6%)
Other less commonly reported movement disorders	
Opsoclonus-myoclonus	1 (2%)
Propiospinal myoclonus	1 (2%)
Paroxysmal dyskinesia	1 (2%)
Myorhythmia	1 (2%)
Myokymia	1(2%)
**Demographics**	
Female to male ratio	7:2
Mean age (SD), in years	44.6 (22.7)
**Types of Publications**	
Case reports	30
Case series	8
Retrospective observational studies	9
Prospective pilot study	1
**Year of Publication**	
Range	1966–2018
**Number of Publications per Decade**	
Until 1990	5
1991–2000	9
2001–2010	20
2011–2018	14

**Table 2:** Characteristics of the papers included in the review.

Movement Disorder	Number of Cases of Patients Published until the Date	Male:Female	Mean Age of Onset (SD)/Age of Onset	Response to GFD E, S, N, L	HLA DQ2/DQ8	CD:GS
Chorea	8	1:7	57.4 (12.9)	E 5 (62.5%) S 2 (25%) L 1 (12.5%)	DQ2(+) 2 (25%) DQ2(-) 2 (25%) NA (50%)	5:3
RLS	65	6:59	NA	E 18 (28%) N 16 (25%) L 31 (47%)	NA	65:0
Myoclonus	28	15:13	47.7 (17.3)	S 1 (3%) N 28 (97%)	NA	28:0
Palatal tremor	3	1:2	51.3 (8.1)	E 1 (33%) N 2 (67%)	DQ2(+) 1 (33%) NA: 2 (67%)	1:2
Dystonia	2	1:1	49.50 (2.1)	N 2 (100%)	NA	2:0
Tremor	9	3:6	54.6 (14.9)	E 6 (67%) N 3 (33%)	DQ2(+) 6 (67%) NA: 3 (33%)	9:0
Parkinsonism	3	0:3	54.0 (18.7)	E 1 (33%) N 2 (67%)	NA	3:0
Tics	1	0:1	13	E (100%)	DQ8(+) 1 (100%)	0:1
OM	1	1:0	2	E (100%)	NA	1:0
PSM	1	0:1	23	E (100%)	NA	1:0
Paroxysmal dyskinesia	1	0:1	0.5	E (100%)	NA	1:0
Myorhythmia	1	0:1	68	N (100%)	DQ2 (+) 1 (100%)	1:0
Myokymia	1	0:1	72	N (100%)	NA	1:0

RLS, restless legs syndrome; OM, opsoclonus-myoclonus; PSM, propiospinal myoclonus; NA, not available; GFD, gluten-free diet; E, evident; S, slight; N, none; L, lack of data; CD, coeliac disease; GS, gluten sensitivity.
